# Bevacizumab for Patients with Recurrent Multifocal Glioblastomas

**DOI:** 10.3390/ijms18112469

**Published:** 2017-11-20

**Authors:** Michael C. Burger, Stella Breuer, Hans C. Cieplik, Patrick N. Harter, Kea Franz, Oliver Bähr, Joachim P. Steinbach

**Affiliations:** 1 Dr. Senckenberg Institute of Neurooncology, Goethe University Hospital, 60528 Frankfurt, Germany; hans.cieplik@kgu.de (H.C.C.); oliver.baehr@med.uni-frankfurt.de (O.B.); joachim.steinbach@med.uni-frankfurt.de (J.P.S.); 2 Institute of Neuroradiology, Goethe University Hospital, 60528 Frankfurt, Germany; stella.breuer@kgu.de; 3 Institute of Neurology (Edinger Institut), Goethe University Hospital, 60528 Frankfurt, Germany; patrick.harter@kgu.de; 4 Department of Neurosurgery, Goethe University Hospital, 60528 Frankfurt, Germany; kea.franz@kgu.de

**Keywords:** glioblastoma, multifocal, bevacizumab, anti-angiogenic therapy, invasive growth, infiltration

## Abstract

In patients with glioblastoma, antiangiogenic therapy with bevacizumab (BEV) has been shown to improve progression-free survival (PFS), but not overall survival (OS). Especially in patients with an unusual infiltrative phenotype as seen in multifocal glioblastoma, the use of BEV therapy is still more controversial. Therefore, we prepared a retrospective case series with 16 patients suffering from a multifocal glioblastoma treated with BEV. We compared these patients to a matched control cohort of 16 patients suffering from glioblastoma with a single lesion treated with BEV. The objective of this study was to evaluate whether the course of disease differs in glioblastoma patients with a multifocal disease pattern compared to those with a single lesion only. Patients were treated with BEV monotherapy or BEV in combination with irinotecan or lomustine (CCNU). Response rates and PFS were similar in both groups. There was a trend for an unfavorable OS in the patient group with multifocal glioblastoma, which was expected due to the generally worse prognosis of multifocal glioblastoma. We investigated whether BEV therapy affects the invasive growth pattern as measured by the appearance of new lesions on magnetic resonance imaging (MRI). Under BEV therapy, there was a trend for a lower frequency of new lesions both in multifocal and solitary glioblastoma. Based on these results, BEV therapy at relapse appears to be justified to no lesser extent in multifocal glioblastoma than in solitary glioblastoma.

## 1. Introduction

Glioblastoma (GB) is characterized by a highly infiltrative growth pattern. Already at the time of diagnosis, glioblastoma cells can be found in parts of the brain distant to the main contrast enhancing tumor bulk [1]. This infiltrative growth pattern increases in later stages of the disease. In autopsy series, a high proportion of patients with GB show a generalized distribution [2]. Multifocal glioblastomas (mfGB) are characterized by several tumor localizations already present at the time of initial diagnosis. Molecular characterization of mfGB has shown that they resemble solitary glioblastomas (sGB), which only have one lesion. However, some aberrations are found more frequently, while others are rare in mfGB as compared to sGB. EGFR amplifications, CDKN2A/B homozygous deletions, and a CYB5R2 overexpression are more frequent in mfGB than in sGB. Conversely, isocitrate dehydrogenase 1 (IDH1), ATP-dependent helicase (ATRX), or platelet-derived growth factor receptor A (PDGFRA) mutations are unusual in mfGB [3,4]. Compared to sGB, patients with mfGB have a worse prognosis and multifocality is an independent unfavorable prognostic factor in GB [3,5]. Contrast-enhancing nodules of mfGB usually derive from the same population of highly infiltrative tumor-initiating cells. The tumor cells infiltrate the brain, proliferate, and subsequently form tumor nodules at various localizations [3]. Initially these nodules usually are not contrast-enhancing. However, as the glioblastoma cells proliferate, their density in the brain tissue increases. From a certain size, the tumor nodules become more and more dependent on neoangiogenesis [6]. The glioblastoma cells secrete vascular endothelial growth factor A (VEGF-A), which induces neovascularization and the formation of a highly dysfunctional vascular system in the tumor. The blood-brain-barrier of these newly formed vessels is impaired as well, leading to a focal cerebral edema [7].

Bevacizumab (BEV) is a monoclonal humanized antibody of the immunoglobulin G1 (IgG1) subtype targeted against VEGF-A. It inhibits the neovascularization through blocking the VEGF signaling. In the existing highly abnormal tumor vasculature, a partial normalization can be observed. Vascular permeability is reduced, and therefore the focal edema is reduced [8]. Vascular normalization initially leads to an increased tumor perfusion as well. Thereby, and through the reduction of the tumor edema, oxygenation of the tumor tissue is improved [9]. However, the ongoing effect of BEV eventually leads to the regression of the tumor vasculature. This results in reduced perfusion and therefore increased hypoxia in the tumor tissue [10]. The hypoxia potentially may push the tumor cells to switch back to a more infiltrative phenotype which is less dependent on angiogenesis [6]. This effect has been observed in mouse xenograft GB models treated with BEV. Under BEV therapy, the glioblastoma cells were characterized by a more infiltrative growth pattern infiltrating neighboring brain areas, and the resulting tumors had a tendency towards a multifocal tumor phenotype. Growing cells dispersed within the brain tissue were supplied with oxygen and nutrients by the existing physiologic brain vasculature [11]. Two uncontrolled patient series seemed to confirm the concern that BEV therapy may increase the invasive phenotype of GB [12,13]. However, these results were not confirmed and BEV was shown not to increase GB invasiveness [14].

In clinical practice, BEV therapy is nevertheless often employed more hesitantly in mfGB than in sGB. One of the main reasons for the caution applied using BEV therapy on mfGB patients is the fear that BEV may further increase the invasive tumor phenotype [15]. This has not been changed by the fact that this concern is based on preclinical data [11] and uncontrolled case series [12,13] that have not been confirmed [14]. Another objection is that mfGB are often characterized by a higher proportion of diffuse infiltrative tumor tissue and might depend to a lesser extent on angiogenic growth and thereby on VEGF-A compared to classical sGB.

In summary, the use of BEV in mfGB is still a very controversial issue, even more so than in sGB. BEV is used in GB as a second-, third-, or fourth-line therapy, often in situations where no other meaningful therapy options are available [16]. It reduces the peritumoral edema and the compression of adjacent areas of the brain. As a result, in many patients neurological symptoms are mitigated. This supportive aspect of BEV therapy conveyed through its anti-edema effect is highly relevant, leading to a transient improvement of patients’ quality of life in the short remaining lifespan [16]. Also, several studies have shown that progression-free survival (PFS) is extended [17,18]. Both effects can be meaningful for patients, as well as their relatives and caregivers. However, large clinical studies have failed to show an increase in overall survival (OS) [19,20].

The question of whether BEV therapy should be applied or not is of great importance for patients who do not have any further therapy options left. BEV is often withheld from mfGB patients, even when there is no therapeutic alternative available and “best supportive care” is the only alternative. Therefore, we performed a retrospective data analysis of the GB patients treated with BEV at our institution.

All patients with mfGB treated with BEV between April 2008 and March 2016 were compared to a matched control cohort with sGB. All patients of the series received BEV therapy in the relapse situation (after the first, second, or third relapse). We compared the response rate, PFS, OS, and the relapse pattern of mfGB patients to sGB patients under BEV therapy. If the objections discussed above would be applicable, the response rate would be lower in mfGB as compared to sGB patients and the amount of new distant tumor locations which occur under BEV therapy would increase.

## 2. Results

**Figure 1:**
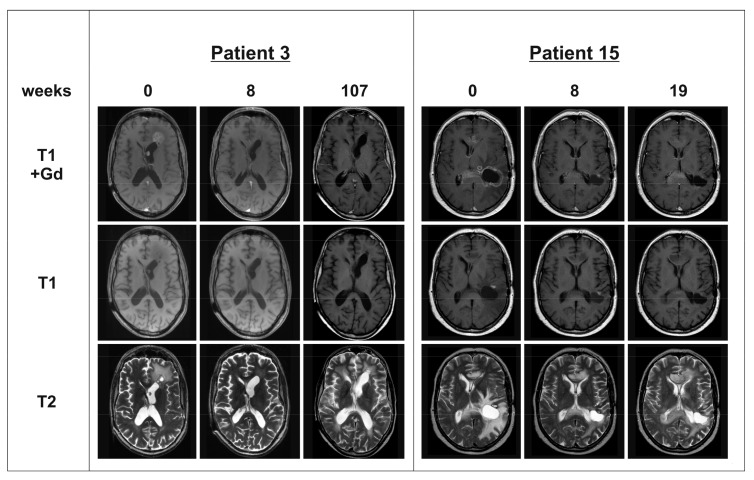
Magnetic resonance imaging (MRI) of patients 3 and 15: **T1** sequences with and without Gadolinium (Gd) contrast enhancer and **T2** sequences were obtained at baseline, follow-up at 8 weeks after Bevacizumab (BEV) therapy initiation and at relapse. Patient 3 achieved partial response (PR) under BEV therapy combined with irinotecan. At week 107, patient 3 showed a progressive contrast enhancement in the area of the septum pellucidum. Patient 15 reached PR under BEV monotherapy. At week 19, both contrast-enhancing lesions (in the anterior and posterior part of the corpus callosum) progressed.

**Figure 2:**
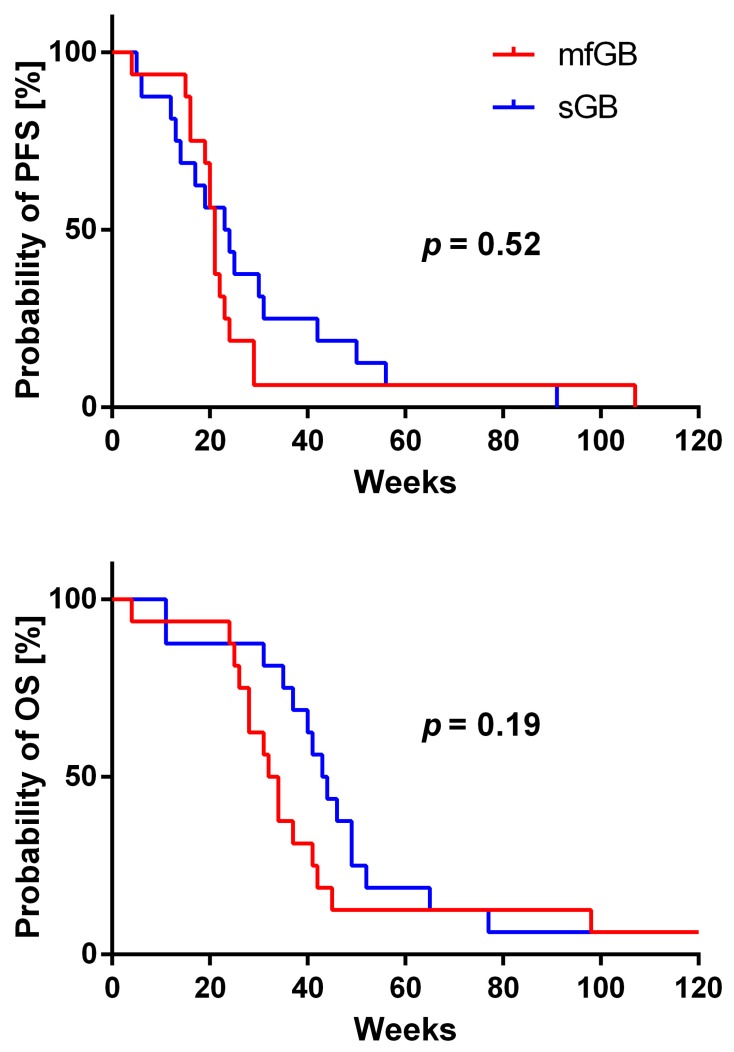
Progression-free survival (PFS) and overall survival (OS). No significant difference from patients with multifocal glioblastomas (mfGB) compared to patients with solitary glioblastomas (sGB) was observed. However, there was a clear trend for worse OS in patients with mfGB (*p* = 0.19).

In the patient group with mfGB, we observed as best response a partial response (PR) in 11 patients, stable disease (SD) in two patients, mixed response (MR) in one patient, and progressive disease (PD) in two patients (see Table 1 and Figure 1). In the sGB matched control cohort, best response was PR in nine patients, SD in three patients, MR response in one patient, and PD in three patients (see Table 2). There was no significant difference in PFS and OS between patients with mfGB and sGB. However, there was a clear trend for shorter OS in mfGB as compared to sGB (see Figure 2). Median PFS was 21 weeks for patients with mfGB, and 23.5 weeks for their matched controls with sGB. Median OS was 33 weeks in the mfGB group, and 43.5 weeks in the sGB group. One patient in each group was still alive at the time of data analysis (patient 3 at week 313 and patient C16 at week 129). In the mfGB group, Karnofsky performance score (KPS) improved after initiating BEV therapy in five patients, stabilized in 10 patients, and deteriorated in one patient. In the matched control group of sGB patients, KPS improved in four patients, stabilized in 10 patients, and deteriorated in two patients. In the mfGB group, steroid intake was reduced after BEV therapy initiation in 13 patients and was left untouched in three patients. In no mfGB patient did the steroid dose have to be increased. In the sGB control cohort, steroid intake was reduced in 12 patients, left unchanged in three patients, and had to be escalated in one patient. To evaluate if the tumors developed an even more infiltrative phenotype under BEV therapy, we explored the share of new lesions (T1 contrast enhancing separated by at least 1 cm from existing lesions) on the last magnetic resonance imaging (MRI) showing progression before the start of BEV therapy and at progression under BEV therapy. In the mfGB goup, new lesions were observed in 40% prior to BEV therapy initiation and in 23.1% of the relapses under BEV therapy (*p* = 0.36). In the sGB control cohort, new lesions occurred in 31.3% prior to and in 21.4% under BEV therapy (*p* = 0.56; see Table 3). There was no significant difference in the frequency of new lesions between the mfGB and sGB groups (*p* = 0.62 prior to BEV therapy; *p* = 0.92 under BEV therapy).

## 3. Discussion

This patient series shows that clinical response on BEV treatment is similar in patients with mfGB as compared to sGB. Response rates, clinical improvement, and the rate of steroid reduction were similar in both groups. In particular, PFS was similar in patients with mfGB as compared to the matched control patients with sGB. There was, however, a trend for a shorter OS in the patients with mfGB. This was not unexpected, as several large studies have shown that BEV therapy does not improve OS [17,19,20] and mfGB per se have a more unfavorable prognosis [3,5]. Therefore, even provided that BEV has a similar effect in mfGB and sGB, OS cannot be expected to be comparable in the two groups. In both the mfGB and sGB groups, we did not observe the development to a more invasive phenotype as measured by the frequency of appearance of new lesions on MRI under BEV therapy. There was even a trend for fewer new lesions both in mfGB and sGB under BEV therapy, which did not reach significance. Comparably, BEV therapy did not result in a more invasive growth pattern in glioma patients with a gliomatosis cerebri growth pattern or leptomeningeal metastases [21,22].

These data show that BEV had a similar effect in the mfGB and sGB groups. Meaningful clinical responses were observed in a similar frequency in both groups. Due to the known prolongation of PFS, the clinical stabilization, and the steroid-sparing effects of BEV in GB, this therapy should be applied in cases where no reasonable therapy alternative is available [17,18,19,20]. These patients often are in a critical condition and further clinical deterioration would make many patients considerably dependent on caregivers. Delaying this deterioration is of high concern both to the patients and their caregivers, even if no prolongation of survival can be achieved. By being able to reduce the steroid dose, typical steroid side effects like osteoporosis, stomach ulcer, and metabolic syndrome can be avoided. On the other hand, possible serious complications of BEV therapy like hypertonia, bowel perforation, necrotizing fasciitis, impaired wound healing, and serious vascular events have to be considered as well [19]. Unfortunately, a direct comparison of patients with mfGB treated with BEV to those not treated with BEV was not possible. When BEV therapy is considered, the choice is usually between BEV therapy and “best supportive care” (BSC). Those patients in a reasonable clinical condition (KPS ≥ 60%) usually received BEV, while the others usually received BSC. Furthermore, the data is limited by the relatively small patient number and their heterogenic pretreatment. Moreover, many patients received concomitant treatment with lomustine (CCNU) or irinotecan. In some patients treated at the beginning of the screening period, *O*-6-methylguanine-DNA-methyltransferase (MGMT) methylations status was not determined. BEV therapy was tolerated well overall; however, due to the retrospective nature of this study, a systematic recording of adverse events was not possible. As this is a retrospective study, no patient recorded outcomes were surveyed. This limitation is highly relevant, as this approach may yield data on the effects of symptom reduction and steroid intake on quality of life (QoL). To overcome the limitations of this retrospective study, a prospective randomized study comparing BEV therapy to BSC, both for mfGB and sGB patients, would be desirable. To avoid confounders, BEV therapy should be examined as a monotherapy. To quantify the results on QoL, a dedicated survey of patient recorded outcomes should be included as well. To reach a sufficient number of patients, a multi-centric approach would be indispensable.

In summary, BEV therapy should not be withheld from mfGB patients solely on the basis of multifocal disease distribution. A transient clinical improvement or at least stabilization could be achieved with BEV therapy in a relevant amount of patients.

## 4. Patients and Methods

A total of 164 patients with GB were treated with BEV alone or in combination with other substances at our center between April 2008 and March 2016. We included all patients exhibiting a multifocal distribution with clearly separated (with at least 1 cm distance) contrast enhancing tumor areas. We identified 16 consecutive patients with recurrent mfGB treated with BEV. All patients were treated with BEV (10 mg/kg IV every other week) as a single agent, or in combination with lomustine (CCNU) or irinotecan (see Table 4). The therapy lines prior to BEV therapy initiation (first-, second-, and/or third-line therapy) are delineated in Table 4 and Table 5 under “pretreatment”. In some patients from the beginning of the screening period the MGMT promoter status was not determined. We selected a matched control cohort of patients with recurrent sGB treated with BEV during the same interval at our institution with similar pretreatment, Karnofsky performance score (KPS), and dose of steroid co-medication at the start of BEV therapy (see Table 5). PFS and OS were illustrated using the Kaplan-Meier estimator. We calculated the significance of the survival analyses with the Log-rank (Mantel-Cox) test. Significance of the proportion of new lesions on imaging was calculated using a two-sided *t*-test. Patient characteristics in both groups were similar overall (see Table 6). However, due to the inherent multifocal tumor distribution, partial or total resection as compared to biopsy only at the time of initial diagnosis was more frequent in sGB than in mfGB (81% vs. 56%). Some patients of this case series were included in previously published studies on BEV therapy in recurrent glioblastoma [23,24].

### O-6-methylguanine-DNA-methyltransferase (MGMT) Promoter Methylation Status Assessment

Vital parts of tumor material were selected for performing the methylation-specific polymerase (MSP) chain reaction. From each paraffin block, four slides, each with a thickness of 10 μm, were cut and vital tumor tissue was punched out. Subsequently, tumor slides were deparaffinized and washed with xylene and 2 × 96% alcohol. Tumor DNA was isolated utilizing the DNeasy Blood & Tissue Kit (Quiagen, Hilden, Germany). We determined nucleic acid concentration with an ultraviolet spectrophotometric analysis. DNA was incubated with sodium bisulfite utilizing the EZ DNA Methylation Gold Kit (Zymo Research, Irvine, CA, USA). We performed the PCR run on a Thermocycler T3000 (Biometra, Göttingen, Germany). We used the following primers: (I) MGMT-methylated forward primer: GTT TTT AGA ACG TTT TGC GTT TCG AC; (II) MGMT-methylated reverse primer: CAC CGT CCC GAA AAA AAA CTC CG; (III) MGMT-unmethylated forward primer: TGT GTT TTT AGA ATG TTT TGT GTT TTG AT; (IV) MGMT-unmethylated reverse primer: CTA CCA CCA TCC CAA AAA AAA ACT CCA, the MSP generates a 122-base pair fragment for the methylated *MGMT* sequence, and a 129-base pair for the unmethylated *MGMT* sequence. For the methylated *MGMT* promoter, DNA from the glioma cell line LNT-229 was applied as a positive control. We utilized DNA isolated from blood obtained from a healthy volunteer donor as a positive control for the unmethylated *MGMT* promoter status. H_2_O was used as a negative control.

This retrospective analysis was approved by the institutional ethics committee of the University Hospital Frankfurt, and all patients gave their written informed consent permitting scientific work with clinical data and MRI scans (reference number 04/09-SNO 01/09).

## 5. Conclusions

BEV has similar effects in patients with mfGB as compared to patients with sGB. Therefore, BEV should not be detained from patients solely on the basis of multifocal tumor distribution. To quantify the effect of BEV both in mfGB and sGB patients, a prospective randomized study comparing BEV therapy to BSC is warranted.

## Figures and Tables

**Table 1:** Outcome of patients with mfGB.

Pat. No.	Combination Therapy	Karnofsky Performance Score (KPS)		Steroid Intake (mg of Dexamethasone per Day)		Best Response (RANO Criteria)	PFS (Weeks)	OS (Weeks)
		At Start of Therapy	Development under Therapy	At Start of Therapy	Under Therapy			
**1**	none	80	−30	0	0	PD	4	4
**2**	CCNU	70	+10	0	0	PD	22	28
**3**	Iri	90	0	4	0	PR	107	n.r.
**4**	none	70	0	6	4	PR	21	32
**5**	Iri	70	0	8	1.5	PR	16	42
**6**	none	80	0	8	1	PR	20	34
**7**	CCNU	90	+10	4	0	SD	20	41
**8**	none	90	0	0	0	SD	24	98
**9**	none	60	0	2	1	MR	29	31
**10**	Iri	60	0	4	0	PR	29	34
**11**	none	70	+10	4	0	PR	15	45
**12**	CCNU	60	0	8	2	PR	21	26
**13**	CCNU	70	+10	2	1	PR	21	25
**14**	none	50	+10	8	0	PR	23	24
**15**	none	80	0	2	0	PR	19	28
**16**	Iri	80	0	4	0	PR	16	37

CCNU = lomustine; Iri = irinotecan; MR = mixed response; n.r. = not reached; OS = overall survival; Pat. No. = Patient number; PD = progressive disease; PFS = progression-free survival; PR = partial response; SD = stable disease; RANO = response assessment in neuro-oncology.

**Table 2:** Outcome of patients with sGB (control cohort).

Pat. No.	Combination Therapy	Karnofsky Performance Score (KPS)		Steroid Intake (mg of Dexamethasone per Day)		Best Response (RANO Criteria)	PFS (Weeks)	OS (Weeks)
		At Start of Therapy	Development under Therapy	At Start of Therapy	Under Therapy			
**C1**	Iri	80	0	0	0	PR	50	65
**C2**	none	70	−10	0	0	PD	5	11
**C3**	none	90	0	4	2	PR	17	43
**C4**	none	70	0	6	4	PR	19	35
**C5**	Iri	70	+10	8	1	PD	12	49
**C6**	none	80	0	8	4	SD	13	37
**C7**	none	90	0	4	2	MR	14	46
**C8**	CCNU	90	0	0	0	SD	42	52
**C9**	none	60	−20	2	8	PD	6	11
**C10**	none	60	0	4	0	PR	31	44
**C11**	Iri	70	+20	4	0	SD	56	77
**C12**	CCNU	60	+10	8	1	PR	23	41
**C13**	CCNU	70	0	2	1	PR	24	40
**C14**	none	50	0	8	4	PR	30	31
**C15**	none	80	+10	2	1	PR	25	49
**C16**	CCNU	80	0	4	0	PR	91	n.r.

CCNU = lomustine; Iri = irinotecan; MR = mixed response; n.r. = not reached; OS = overall survival; Pat. No. = Patient number; PD = progressive disease; PFS = progression-free survival; PR = partial response; SD = stable disease; RANO = response assessment in neuro-oncology.

**Table 3:** Relapse pattern prior to BEV therapy initiation and under BEV therapy.

Pat. No.	Prior to BEV Therapy Initiation	Under BEV Therapy	Pat. No.	Prior to BEV Therapy Initiation	Under BEV Therapy
**1**	new lesion	not evaluable	**C1**	new lesion	new lesion
**2**	progressive lesion	progressive lesion	**C2**	new lesion	progressive lesion
**3**	progressive lesion	progressive lesion	**C3**	new lesion	progressive lesion
**4**	new lesion	new lesion	**C4**	progressive lesion	progressive lesion
**5**	progressive lesion	progressive lesion	**C5**	new lesion	progressive lesion
**6**	new lesion	progressive lesion	**C6**	progressive lesion	not evaluable
**7**	progressive lesion	progressive lesion	**C7**	progressive lesion	progressive lesion
**8**	new lesion	progressive lesion	**C8**	progressive lesion	progressive lesion
**9**	progressive lesion	progressive lesion	**C9**	progressive lesion	not evaluable
**10**	progressive lesion	progressive lesion	**C10**	progressive lesion	progressive lesion
**11**	progressive lesion	progressive lesion	**C11**	new lesion	new lesion
**12**	progressive lesion	not evaluable	**C12**	progressive lesion	progressive lesion
**13**	new lesion	new lesion	**C13**	progressive lesion	progressive lesion
**14**	not evaluable	new lesion	**C14**	progressive lesion	new lesion
**15**	new lesion	progressive lesion	**C15**	progressive lesion	progressive lesion
**16**	progressive lesion	not evaluable	**C16**	progressive lesion	progressive lesion

Progressive lesion: volume of existing lesions increasing; new lesion: new T1 contrast-enhancing lesion(s); not evaluable: no magnetic resonance imaging done.

**Table 4:** Characteristics of patients with mfGB.

Pat. No.	Age	Gender	MGMT	Pretreatment
**1**	50	F	meth.	S, XRT-TMZ, CCNU, mTMZ
**2**	70	M	meth.	XRT-TMZ, reXRT-TMZ
**3**	50	M	unmeth.	S, XRT, CCNU/TMZ
**4**	33	M	n.d.	S, XRT-TMZ, reXRT, CCNU/VM26
**5**	49	M	n.d.	XRT-TMZ, TMZ 7-14
**6**	47	M	unmeth.	XRT-TMZ, CCNU, reXRT
**7**	51	F	meth.	S, XRT-TMZ, CCNU
**8**	55	M	unmeth.	S, XRT-TMZ
**9**	49	F	unmeth.	S, XRT-TMZ, CCNU/VM26
**10**	46	M	n.d.	S, XRT-TMZ, TMZ 7-14
**11**	56	M	n.d.	XRT-TMZ, TMZ 7-14
**12**	60	M	meth.	XRT-TMZ, reXRT
**13**	61	M	unmeth.	XRT-TMZ
**14**	65	M	unmeth.	XRT
**15**	58	M	n.d.	S, XRT-TMZ
**16**	62	M	meth.	S, XRT-TMZ, CCNU/TMZ

CCNU = lomustine; CCNU/TMZ = lomustine/temozolomide; CCNU/VM26 = lomustine/teniposide; F = female; M = male; meth. = *O*-6-methylguanine-DNA-methyltransferase (MGMT) promotor hypermethylation; mTMZ = metronomic temozolomide scheme (“always on”); n.d. = MGMT promotor status not determined; Pat. No. = Patient number; reXRT = relapse radiotherapy; reXRT-TMZ = relapse radiotherapy with concomitant and adjuvant temozolomide; S = surgery; TMZ = temozolomide 5/28; TMZ 7–14 = dose dense temozolomide scheme (“one week on/one week off”); unmeth. = no MGMT promotor hypermethylation; XRT = radiotherapy; XRT-TMZ = radiotherapy with concomitant and adjuvant temozolomide (in accordance with EORTC 26981).

**Table 5:** Characteristics of patients with sGB (control cohort).

Pat. No.	Age	Gender	MGMT	Pretreatment
C1	65	F	meth.	S, XRT-TMZ, CCNU-TMZ, reXRT
C2	69	M	n.d.	XRT-TMZ, TMZ 7-14
C3	69	F	unmeth.	S, XRT-TMZ, TMZ 7-14
C4	31	M	n.d.	S, XRT-TMZ, reS, TMZ 7-14, CCNU
C5	48	M	unmeth.	XRT-TMZ, TMZ 7-14
C6	66	M	meth.	S, XRT-TMZ, TMZ, TMZ 7-14
C7	49	M	unmeth.	S, XRT-TMZ, CCNU
C8	29	M	n.d.	S, XRT-TMZ
C9	52	M	unmeth.	S, XRT-TMZ, TMZ 21-28
C10	48	F	meth.	S, XRT-TMZ, reXRT-TMZ
C11	54	F	n.d.	S, XRT-TMZ, TMZ 7-14
C12	58	M	n.d.	S, XRT-TMZ, reS, TMZ 7-14
C13	52	M	n.d.	S, XRT-TMZ
C14	48	F	unmeth.	S, XRT
C15	63	F	unmeth.	XRT-TMZ
C16	50	M	meth.	S, XRT-TMZ, CCNU

CCNU = lomustine; CCNU/TMZ = lomustine/temozolomide; CCNU/VM26 = lomustine/teniposide; F = female; M = male; meth. = MGMT promotor hypermethylation; mTMZ = metronomic temozolomide scheme (“always on”); n.d. = MGMT promotor status not determined; Pat. No. = Patient number; reS = relapse surgery; reXRT = relapse radiotherapy; reXRT-TMZ = relapse radiotherapy with concomitant and adjuvant temozolomide; S = surgery; TMZ = temozolomide 5/28; TMZ 7–14 = dose dense temozolomide scheme (“one week on/one week off”); TMZ 21-28 = dose dense temozolomide scheme (“three weeks on/one week off”); unmeth. = no MGMT promotor hypermethylation; XRT = radiotherapy; XRT-TMZ = radiotherapy with concomitant and adjuvant temozolomide (in accordance with EORTC 26981).

**Table 6:** Comparison of patient characteristics.

Patient Characteristics	mfGB	sGB
Female/male patients	3/13	6/10
Median patients’ age at BEV therapy initiation (years)	53	53.5
Surgery/biopsy at diagnosis	9/7	13/3
MGMT meth./unmeth./n.d.	5/6/5	4/6/6
Median number of previous chemotherapy lines	2	2
Median KPS at BEV therapy initiation	70	70
Median steroid intake at BEV therapy initiation (mg of dexamethasone per day)	4	4

BEV = bevacizumab; KPS = Karnofsky performance score; meth. = MGMT promotor hypermethylation; n.d. = MGMT promotor status not determined; unmeth. = no MGMT promotor hypermethylation.

## Abbreviations

ATRX	ATP-Dependent Helicase
BEV	Bevacizumab
BSC	Best Supportive Care
CCNU	Lomustine
CCNU/TMZ	Lomustine/Temozolomide
CDKN2A/B	Cyclin-Dependent Kinase Inhibitor 2A/B
CYB5R2	Cytochrome b5 Reductase 2
DNA	Deoxyribonucleic Acid
EGFR	Epidermal Growth Factor Receptor
F	Female
GB	Glioblastoma
Gd	Gadolinium contrast enhancer
IDH1	Isocitrate Dehydrogenase 1
Iri	Irinotecan
KPS	Karnofsky Performance Score
M	Male
meth.	MGMT Promotor hypermethylation
mfGB	Multifocal Glioblastoma
MGMT	*O*-6-Methylguanine-DNA-Methyltransferase
MR	Mixed Response
MRI	Magnetic Resonance Imaging
MSP	Methylation-Specific Polymerase Chain Reaction
mTMZ	Metronomic Temozolomide Scheme (“Always On”)
n.d.	Not Determined
n.r.	Not Reached
OS	Overall Survival
Pat. No.	Patient Number
PD	Progressive Disease
PDGFRA	Platelet-Derived Growth Factor Receptor A
PFS	Progression-Free Survival
PR	Partial Response
QoL	Quality of Life
reS	Relapse Surgery
reXRT	Relapse Radiotherapy
reXRT-TMZ	Relapse Radiotherapy with Concomitant and Adjuvant Temozolomide
S	Surgery
SD	Stable Disease
sGB	Solitary Glioblastoma
TMZ	Temozolomide 5/28
TMZ 7-14	Dose Dense Temozolomide Scheme (“One Week On/One Week Off”)
TMZ 21-28	Dose Dense Temozolomide Scheme (“Three Weeks On/One Week Off”)
unmeth.	No MGMT Promotor Hypermethylation
VEGF	Vascular Endothelial Growth Factor
VM26	Teniposide
XRT	Radiotherapy
XRT-TMZ	Radiotherapy with Concomitant and Adjuvant Temozolomide
